# Cutaneous manifestations in a patient with chronic lymphocytic leukemia involving the head, neck and distal extremities

**DOI:** 10.3892/etm.2015.2178

**Published:** 2015-01-13

**Authors:** CHONGRONG LU, LI LI, QIAOHUA QIAO, GUOZHEN LIU, LIZHENG FANG

**Affiliations:** 1 Department of Family Medicine, Sir Run Run Shaw Hospital, School of Medicine, Zhejiang University, Hangzhou, Zhejiang 310016, P.R. China; 2 Family Medicine Residency Program, Genesys Regional Medical Center, Grand Blanc, MI 48439, USA

**Keywords:** chronic lymphocytic leukemia, infiltrative, skin

## Abstract

Chronic lymphocytic leukemia (CLL) infiltrating the skin is uncommon and can present in many different ways. The present study reports a case of CLL infiltrating multiple body areas. A 57-year-old male with a 10-year history of subclinical B-cell chronic lymphocytic leukemia (B-CLL) presented with skin hypertrophic changes of the ears, eyebrows, tip of the nose, toes and fingers. In addition, the patient had erythematous plaques on the buttocks. Histopathology revealed a lymphocytic infiltrate. The patient rejected the recommended chemotherapy and, following a three-year follow-up, remained alive with mildly aggravated symptoms. It has previously been reported that infiltrative CLL can involve the head and neck; however, involvement of multiple body areas, particularly toes and fingers is rare. This case highlights the importance of considering leukemia cutis in patients with underlying CLL who present with unusual clinical features.

## Introduction

B-cell chronic lymphocytic leukemia (B-CLL) is the most common form of leukemia and occurs with a male predominance (1), with majority of patients being over the age of 45 years. Medical literature describing the appearance of cutaneous involvement in patients with B-CLL is limited. In the majority of cases, the cutaneous lesions are nonspecific manifestations associated with an impaired immune system (2). The reported specific skin lesions include nodules, papules, infiltrates, plaques, ulcerations and exfoliative erythroderma (2–5), presenting predominantly in the head and neck areas. In the present study, a 57-year-old man with B-CLL who presented with plaque skin infiltrates affecting the limbs, buttocks and prominent parts of the face is described.

## Case report

A 57-year-old male presented with a one-week history of non-pitting edema in the hands and feet in addition to erythematous skin on both buttocks. The patient had a 10-year history of untreated B-CLL and self-reported concurrent gradual hypertrophic changes of the ears, eyebrows, nose and toes. Informed consent was obtained from the patient.

Blood tests showed a percentage of blood lymphocytes of 65.4% (normal, 20–45%), a lymphocyte count of 5.7×10^9^/l (normal, 1.5–3×10^9^/l) and no significant abnormalities in erythrocyte sedimentation rate, tumor markers and biochemistry. A 24-h electrocardiogram showed atrial flutter and atrial fibrillation. The patient was admitted to Sir Run Run Shaw Hospital, School of Medicine, Zhejiang University (Hangzhou, China) for further diagnosis and treatment.

**Figure 1:**
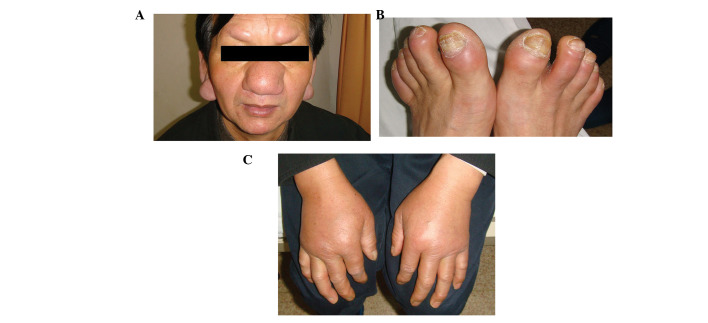
Appearance of the patient. Chronic lymphocytic leukemia infiltration of (A) the ears, eyebrows and nose and (B) the toes. (C) Non-pitting edema bilaterally on the back of the hands.

On examination, the vital signs were stable. The patient was observed to have plum-colored swelling involving the prominent parts of the ears (helix, tragus and ear lobe), the eyebrows, nose and toes and non-pitting edema on the dorsal surfaces of the hands and fingers (Fig. 1). The lymph nodes in the right submandibular, left subclavian, left axilla and groin areas were enlarged. Non-blanching erythematous plaques were present on the patient’s buttocks.

**Figure 2:**
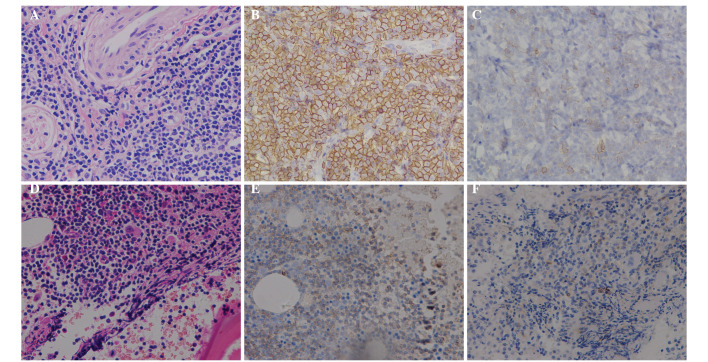
Biopsy results. (A) Histological examination of the right auricular lobule showed atypical hyperplasia of the lymphoid tissue (magnification, ×400). Immunohistochemistry of the right auricular lobule revealed (B) CD20^+^ and (C) CD5^+^ cells (magnification, ×400). (D) Hematoxylin and eosin staining of bone marrow revealed the diffuse infiltration of small lymphocytic cells (magnification, ×400). Immunohistochemistry of bone marrow revealed (E) CD20^+^ and (F) CD23^+^ cells (magnification, ×400).

Laboratory findings when the patient was first admitted were as follows: The leukocyte count was 14.0×10^9^/l (normal range, 4.0–10.0×10^9^/l), the lymphocyte count was 10.6×10^9^/l (normal range, 1.5–3.0×10^9^/l), the percentage of lymphocytes was 65.4% (normal range, 20–45%), the neutrophil count was 2.9×10^9^/l (normal range, 1.5–3.0×10^9^/l) and the percentage of neutrophils was 20.5% (normal range, 55–75%). Histopathology of a biopsy from the right auricular lobule showed atypical hyperplasia of lymphoid tissue. Immunohistochemical investigation revealed that the right auricular lobule co-expressed CD20 and CD5, which is consistent with CLL/small lymphocytic lymphoma. Hematoxylin and eosin staining of bone marrow revealed the diffuse infiltration of small lymphocytic cells. Immunohistochemical staining revealed that the bone marrow was positive for the B-cell marker CD20 and also partly positive for CD23 and CD5, which is consistent with an infiltrate of CLL (Fig. 2).

The patient was recommended to receive chemotherapy but declined it due to a poor financial situation and fear of the side-effects of chemotherapy. Following a three-year follow-up in the clinic, the patient remained alive with mildly aggravated symptoms.

## Discussion

B-CLL is a low-grade, B-cell lymphoproliferative monoclonal disorder in which functionally immunoincompetent lymphocytes are progressive accumulated, and thereby affect immune function and normal hematopoiesis. It is associated with an increased incidence of other malignancies, including squamous cell carcinoma, basal cell carcinoma, malignant melanoma and Merkel cell carcinoma (6). B-CLL patients are prone to cutaneous infections, particularly viral infections, and have exaggerated reactions to insect bites (6,7).

From a broader point of view, the incidence of lymphomas, in particular that of B-CLL, is increasing worldwide (8,9). The majority of patients with CLL manifest atypical clinical features. The most common symptoms and signs of this condition include fatigue, fever, easy bruising and generalized lymphadenopathy (10).

Although skin infiltration occurs in 3–50% of patients with leukemias or lymphomas overall, it is rare in patients with CLL (11,12). When evident skin involvement is observed in CLL, it usually is seen in Richter syndrome or T-cell CLL (13,14), which generally indicates a poor prognosis (3,15,16). Medical literature reports that CLL can cause skin infiltrates, chronic and relapsing pruritic skin lesions (17) and indurated plaques of the eyebrows (18). Reports of cutaneous symptoms being the primary manifestation of B-CLL are unusual (3,19,20). The case described in the present study presented as a skin infiltrate involving prominent parts of the head, such as the nose, ears and eyebrows, and other parts of the body, including the fingers and toes, a rare combination which might shed light on the mechanism involved in attracting leukemic cells of CLL patients to the skin. In the present case, this process was not investigated further and the possibilities can only be speculated on. The mechanism of cutaneous infiltration has not been fully elucidated. It has been postulated that skin invasiveness may be caused by the upregulation of intercellular adhesion molecule 1 (ICAM-1) and lymphocyte function-associated antigen 1 (LFA-1) (21). It is important to be aware of the possibility of these unusual presentations of cutaneous B-CLL, although skin involvement in B-CLL may be consistent with prolonged survival (15). Additional investigations into the behavior of B-CLL in the skin may further elucidate how this condition develops.
